# Early and late HIV-1 membrane fusion events are impaired by sphinganine lipidated peptides that target the fusion site

**DOI:** 10.1042/BJ20140189

**Published:** 2014-06-26

**Authors:** Yoel A. Klug, Avraham Ashkenazi, Mathias Viard, Ziv Porat, Robert Blumenthal, Yechiel Shai

**Affiliations:** *Department of Biological Chemistry, Weizmann Institute of Science, Rehovot 7610001, Israel; †Section on Membrane Structure and Function, Basic Research Laboratory, Center for Cancer Research, National Cancer Institute, National Institutes of Health, Frederick, MD 21702, U.S.A.; ‡Basic Science Program, Leidos Biomedical Research, NCI Center for Cancer Research, Frederick National Laboratory for Cancer Research, Frederick, MD 21702, U.S.A.; §Flow Cytometry Unit, Department of Biological Services, Weizmann Institute of Science, Rehovot 7610001, Israel

**Keywords:** biophysics, HIV, HIV entry inhibitor, membrane fusion, membrane protein, protein folding, Chol, cholesterol, CHR, C-heptad repeat, DCM, dichloromethane, DHSM, dihydrosphingomyelin, DIEA, *N*,*N*-di-isopropylethylamine, DMEM, Dulbecco’s modified Eagle’s medium, DMF, dimethylformamide, ENV, envelope glycoprotein gp160, ER, endoplasmic reticulum, FP, fusion peptide, LUV, large unilamellar vesicle, NA, numerical aperture, NBD, 7-nitrobenz-2-oxa-1,3-diazole, NHR, N-heptad repeat, PA, palmitic acid, PC, phosphatidylcholine, PE, phosphatidylethanolamine, Rho, rhodamine, RP-HPLC, reverse-phase HPLC, SHB, six helix bundle, SM, sphingomyelin, TFA, trifluoroacetic acid, XTT, 2,3-bis-(2-methoxy-4-nitro-5-sulfophenyl)-2*H*-tetrazolium-5-carboxanilide

## Abstract

Lipid-conjugated peptides have advanced the understanding of membrane protein functions and the roles of lipids in the membrane milieu. These lipopeptides modulate various biological systems such as viral fusion. A single function has been suggested for the lipid, binding to the membrane and thus elevating the local concentration of the peptide at the target site. In the present paper, we challenged this argument by exploring in-depth the antiviral mechanism of lipopeptides, which comprise sphinganine, the lipid backbone of DHSM (dihydrosphingomyelin), and an HIV-1 envelope-derived peptide. Surprisingly, we discovered a partnership between the lipid and the peptide that impaired early membrane fusion events by reducing CD4 receptor lateral diffusion and HIV-1 fusion peptide-mediated lipid mixing. Moreover, only the joint function of sphinganine and its conjugate peptide disrupted HIV-1 fusion protein assembly and folding at the later fusion steps. Via imaging techniques we revealed for the first time the direct localization of these lipopeptides to the virus–cell and cell–cell contact sites. Overall, the findings of the present study may suggest lipid–protein interactions in various biological systems and may help uncover a role for elevated DHSM in HIV-1 and its target cell membranes.

## Abstract 2

We show that sphinganine lipidated peptides affect membrane fusion, modulate the membrane and disrupt protein assembly. In addition the findings may aid in deciphering the function of DHSM in biological membranes.

## INTRODUCTION

HIV-1 entry into the cells is blocked by alteration in the levels of Chol (cholesterol) and sphingolipids that are essential structural components of the cell membrane [1,2]. Sphingolipids and Chol form assemblies in the cell membrane termed ordered domains [3], which mediate virion release from the cells and entry into them [4–6]. The role and importance of these domains have been elucidated by studies focusing on viral entry [7–9].

HIV-1 enters host cells by membrane fusion, which is facilitated by the trimeric viral envelope glycoprotein [ENV (envelope glycoprotein gp160)] [10,11]. During membrane fusion conformational changes in ENV occur [12], which expose the gp41 FP fusion protein and allow it to extend, thus revealing its N-terminal FP (fusion peptide) (pre-hairpin conformation). Consequently a gp41-central coiled-coil is formed from a trimer of NHR (N-heptad repeat) regions packed into three CHR (C-heptad repeat) regions. This core structure, termed the SHB (six helix bundle) is important for complete membrane fusion and pore formation [13–16]. Disruption of the core formation is an established target for N- and C-peptide fusion inhibitors, which are derived from the NHR and CHR regions respectively [16–20].

The HIV-1 membrane, which is acquired from the host cell through viral release, is enriched with the sphingolipid DHSM (dihydrosphingomyelin) [21]. This enrichment is surprising since DHSM is rare in human cell membranes and is primarily found in the human eye lens [22]. The unique structure of the DHSM lipid backbone consists of a saturated acyl chain that is linked to dihydrosphingosine (sphinganine) [23]. In model membranes, Chol binds DHSM to form more condensed domains than the ones formed with SM (sphingomyelin) [24]. The rarity of DHSM, together with its enrichment in the HIV-1 membrane, suggest the possibility that DHSM plays a role in gp41-mediated membrane fusion.

It has been shown that anti-viral activity can be conferred to otherwise inert short gp41-derived peptides via the conjugation to the lipid base of DHSM, sphinganine (termed sphingopeptides). In addition, the function of the sphingopeptide has been shown to rely on the sequence and identity of the peptide [25]. The mechanism by which sphingopeptides act is unknown. By using functional, structural and advanced imaging techniques, we investigated the relationship between the lipid and the peptide in the context of HIV-1 fusion inhibition and revealed a unique inhibitory effect between the peptide and the sphinganine backbone.

## MATERIALS AND METHODS

### Peptide synthesis, lipid moiety conjugation and fluorescent labelling

Peptides were synthesized on Rink Amide MBHA resin by using the Fmoc strategy as described previously [26]. Several peptides contain a lysine residue at their C-terminus with an MTT side-chain protecting group (Novabiochem) that requires a special deprotection step under mild acidic conditions [2×1 min of 5% TFA (trifluoroacetic acid) in DCM (dichloromethane) and 30 min of 1% TFA in DCM]. This enables the conjugation of a lipid moiety to the C-terminus. Conjugation of hexadecanoic acid [PA (palmitic acid); C_16_; Sigma Chemical) to the C-terminus of selected peptides was performed using standard Fmoc chemistry. Conjugation of D-erythro sphinganine (D-erythro-dihydrosphingosine; Matreya LCC) to the C-terminus of a peptide was performed as follows. First, 20 equivalents of DSC (*N,N*′-disuccinimidyl carbonate; Chem-Impex International) and 20 equivalents of DIEA (*N*,*N*-di-isopropylethylamine) were added to the resin for 2 h in DMF (dimethylformamide). Then, two equivalents of sphinganine and two equivalents of DIEA were added for overnight incubation in DMF anhydrous. Addition of a Rho (rhodamine) [5(6)-carboxy-tetramethylrhodamine] fluorescent probe (Chem-Impex International) to the N- terminus of selected peptides was performed by standard Fmoc chemistry. All peptides were cleaved from the resin by a TFA/DDW (doubly distilled water)/TES (triethylsilane) [93.1:4.9:2 (v/v)] mixture, and purified by RP-HPLC (reverse-phase HPLC) to >95% homogeneity. The molecular mass of the peptides was confirmed by platform LCA (liquid chromatography) ESI–MS (electrospray ionization mass spectrometry).

### Infectivity assay with fully infectious HIV-1

A stock of fully infectious HIV-1 HXB2 concentrated virus was a gift from the AIDS Vaccine Program, SAIC. The infectivity of HIV-1 HXB2 was determined using the TZM-bl cell line as a reporter in the presence of various compounds. Cells were added (2×10^4^ cells/well) to a 96-well clear-bottomed microtitre plate with 10% serum-supplemented DMEM (Dulbecco's modified Eagle's medium). Plates were incubated at 37°C for 18–24 h to allow the cells to adhere. The media were then aspirated from each well and replaced with serum-free DMEM containing 40 μg/ml DEAE-dextran. Stock dilutions of each peptide were prepared in DMSO so that each final concentration was achieved with 1% dilution. Upon addition of the compounds, the virus was added to the cells, incubated at 37°C for 18 h to allow the infection to occur and luciferase activity analysed using the Steady-Glo Luciferase assay kit (Promega). Fitting of the data points was performed according to the following equation, derived from Hills’ equation as described previously [27]:

(1) $$Y\left(x\right)\;=\;B\;x\left(\frac{A^{C}}{X^{C}+A^{C}}\right)$$

where *B* is the maximum value (and therefore equals 100% fusion), *A* is the value of an inhibitory concentration at 50% viral infectivity (IC_50_) and *C* represents Hill's coefficient. For the fitting, we uploaded the *X* and *Y* values of the data into a non-linear least squares regression (curve fitter) program that provided the IC_50_ value (parameter *A*). Using the provided IC_50_ value we further calculated the IC_90_ values using eqn (2):

(2) $$X\;={\left(\frac{A^{C}B}{Y}-A^{C}\right)}^{1/c}$$

### Cell–cell fusion assay

The effector cells used were the ENV-expressing HL2/3 cells, a HeLa-derived cell-line which constitutively expresses the HXB2 strain of the HIV-1 ENV glycoprotein along with the Tat protein. TZM-bl cells were used as the target cells. The fusion of HL2-3 cells with TZM-bl cells was assessed through luciferase expression. The TZM-bl cells were seeded at 2×10^4^ cells/well overnight in a 96-well plate. The medium was then aspirated from each well and replaced with serum-free DMEM containing 40 μg/ml DEAE-dextran. Stock dilutions of each peptide were prepared in DMSO so that each final concentration was achieved with 1% dilution. Upon addition of the peptides, the HL2-3 cells were added to the TZM-bl cells in serum-free DMEM containing 40 μg/ml DEAE-dextran at a 1:1 cell ratio. The cells were co-cultured at 37°C for 6 h to allow the fusion to occur. Luciferase activity was analysed using the Steady-Glo Luciferase assay kit (Promega).

### XTT cytotoxicity assay

Aliquots of 2.5×10^4^ cells were distributed into a 96-well plate in the presence of 100–10 μM of the different compounds for 4 h. Wells in the last two columns served as blanks (medium only), and 100% survival controls (cells and medium only). After incubation, the XTT [2,3-bis-(2-methoxy-4-nitro-5-sulfophenyl)-2*H*-tetrazolium-5-carboxanilide] reaction solution [benzene sulfonic acid hydrate/*N*-methyl dibenzopyrazine methyl sulfate (50:1)] was added for an additional 2 h. Attenuance was read at a 450-nm wavelength in an enzyme-linked immunoabsorbent assay plate reader. The percentage of toxicity was calculated relative to the control, 2.5×10^4^ cells in medium with no peptide added.

### Confocal analysis

HXB2 virus was labelled with the non-exchangeable fluorescent lipid analogue PKH67 (Sigma). Non-incorporated PKH67 was removed through passage of the virus on a PD-10 column (GE Healthcare). TZM cells were plated on 35-mm glass-bottomed four-chamber dishes (In Vitro Scientific). They were incubated with 1.3 μM Rho-labelled sphingosine PBDK or AMP1L for 40 min at room temperature in Opti-MEM® (Life Technologies). The cells were washed twice with Opti-MEM® and the virus was added to the plates that were subjected to spinoculation at 4°C (1940 rev./min for 1 h). The cells were then washed and incubated in Opti-MEM® at 37°C for 40 min at which stage they were fixed with 4% paraformaldehyde for 20 min. The images were collected with a Zeiss LSM 510 (Carl Zeiss) confocal laser-scanning microscope. For PKH67 excitation, we used a 488-nm Ar^+^ laser line at 1.1% intensity. The emission light was collected with a 500–550 bandpass filter. For Rho excitation we used a Helium-Neon 543-nm laser line at 18% intensity. The emission light was collected with a 560-nm-long pass filter. A × 40/1.3 NA (numerical aperture) oil-immersion objective lens was used with a zoom factor of 4. Images were analysed using the Imaris analysing software (Bitplane).

### PKH67 labelling efficacy

TZM-bl cells were lifted in cell dissociation buffer, washed twice with PBS and incubated for 5 min at room temperature with 2 μM PKH67 in diluent C (Sigma). After incubation the cells were washed with PBS and further incubated in 10% serum-supplemented DMEM for 5 min at room temperature. Next the cells were washed again twice with PBS before being submitted to FACS analysis.

### ImageStreamX analysis

TZM-bl target cells and HL2/3 effector cells were harvested separately and stained with Hoechst (2 μl per 5×10^6^ cells) and DiD (5 μl per 3×10^6^ cells) respectively. Stained and unstained cells were then plated separately and left overnight at 37°C. The next day both unstained and stained cells were harvested, counted and diluted to 2×10^6^ cells per 1 ml. In order to initiate the fusion process 10^6^ cells of each distinct cell line were mixed together in a 24-well plate and left for 3 h at 37°C and the queried Rho-labelled peptides were added 30 min before the end of incubation. The cells were fixed in 3% paraformaldehyde in order to preserve fragile fusion events. Images were compensated for fluorescent dye overlap by using single-stain controls. Cells were gated for single cells or doublets using the area and aspect ratio features, and for focused cells using the Gradient RMS feature as described previously [28]. Co-localization of the fully infectious virus and each peptide was determined using the similarity feature, which calculates the log-transformed Pearson's correlation coefficient between the two stainings, on a pixel by pixel basis. Localization of the peptide during fusion events was measured using the max contour position feature, which calculates the location of the highest intensity concentration of the staining relative to the entire cell mask (this was done on a threshold mask which takes the top 80% intensity pixels, to eliminate staining background noise). Values closer to 1 represent the periphery of the doublet and closer to 0 the fusion interface.

### Determination of secondary structures

CD measurements were performed by using an Applied Photo physics spectropolarimeter. The spectra were scanned using a thermostatic quartz cuvette with a pathlength of 1 mm. Wavelength scans were performed at 25°C; the average recording time was 7 s, in 1-nm steps and at a wavelength range of 190–260 nm; and recordings were done in triplicate. Each peptide concentration was 10 μM in Hepes buffer (5 mM, pH 7.4).

### Native PAGE

Native gel electrophoresis was carried out by using an 18% polyacrylamide gel. Peptide samples were dissolved in PBS such that N36 and the disrupting agent were mixed before C34 addition. The peptides were then heated for 30 min at 37°C to enhance C34 to N36 binding. N36, C34 and the disrupting agent were loaded on the gel at the final ratio of 1:1:0.5 respectively with a total protein of 32–42 μg depending on the disrupting agent. The gel was subjected to Coomassie Blue staining for protein detection.

### Preparation of lipid vesicles and lipid mixing assay

LUVs (large unilamellar vesicles) were prepared as described previously [29] from egg PC (phosphatidylcholine), Chol and egg yolk SM (Sigma Chemical). A dried film of lipids containing a total of 3 mg of PC/SM/Chol (1:1:1) was suspended in PBS and vortex-mixed for 1.5 min. The lipid suspension underwent five cycles of freezing–thawing and then extrusion through polycarbonate membranes with 1-μm and 0.1-μm diameter pores 21 times. Pre-labelled LUVs PC/SM/Chol (1:1:1) [0.6 mole percent NBD (7-nitrobenz-2-oxa-1,3-diazole)–PE (phosphatidylethanolamine) and 0.6 mole percent Rho–PE each] were constructed (as described above) and added together with the unlabelled LUVs to a PBS−/− solution (no Ca^2+^ or Mg^2+^), where the labelled LUVs constituted 10% of the LUV concentration and the unlabelled LUVs 90%, reaching an LUV final concentration of 100 μM. All fluorescence measurements were performed on a SLM-AMINCO Bowman series 2-luminescence spectrometer at 25°C. LUVs were pre-incubated before fluorescent reading with various lipids at a concentration of 12.5 μM. All measurements were performed in a quartz cuvette with constant magnetic stirring. Lipid mixing by the FP_1-33_ (HXB2 strain 512-544) was evaluated by the increase in NBD fluorescence, the energy donor, at 530 nm which was monitored with the excitation set at 467 nm. The FP was added in several sequential doses after pre-incubated LUVs reached a plateau. Data were collected for several additional minutes after FP addition to ensure a steady state indicated by a plateau. The fluorescence increase after the addition of Triton X-100 (0.05% v/v) was referred to as 100% lipid mixing.

### Assessment of CD4 diffusion through the cell membrane via fluorescence recovery after photobleaching

TZM-bl cells transfected with the CD4–GFP construct [a gift from Dr Waldemar Popik (Meharry Medical College, Nashville, TN, U.S.A.)] and described previously [30]. A total of 40 data points were acquired for image analysis. Data from the whole cell were analysed using the 1D FRAP model [31]. To perform the FRAP experiments, we used a Zeiss LSM 510 confocal laser-scanning microscope. TZM-bl cells were plated on to 35-mm glass-bottomed four-chambered dishes. The experiments were carried under physiological conditions of 37°C and 5% CO_2_ in a stage incubation system (Incubator S; PeCon). For GFP excitation, we used a 488 nm Ar+ laser line at 1% intensity. The emission light was collected with a 500–550 nm bandpass filter. A × 40/1.3 NA oil-immersion objective lens was used with a zoom factor of 4. A 40×20 pixels (4.4×2.2 μM) bleach region was drawn perpendicular to the plasma membrane. The laser output was kept at 1% during image acquisition to limit inadvertent photobleaching, which was assessed through four images taken pre-bleach. Photobleaching was performed by increasing the laser output to 100% for 50 iterations. Image analysis was performed using the MIPAV (Medical Imaging Processing, Analysis, and Visualization; [32]) software package. Data were corrected with background subtraction, as well as normalization for the inadvertent photobleaching rate calculated from the whole cell.

### Statistical analysis

A one-tailed Student's *t* test or Mann–Whitney *U* test were used as appropriate. *P*<0.05 was considered significant. Results are shown as means±S.E.M.

## RESULTS

### PBDK–sphing inhibits HIV infectivity

**Figure 1:**
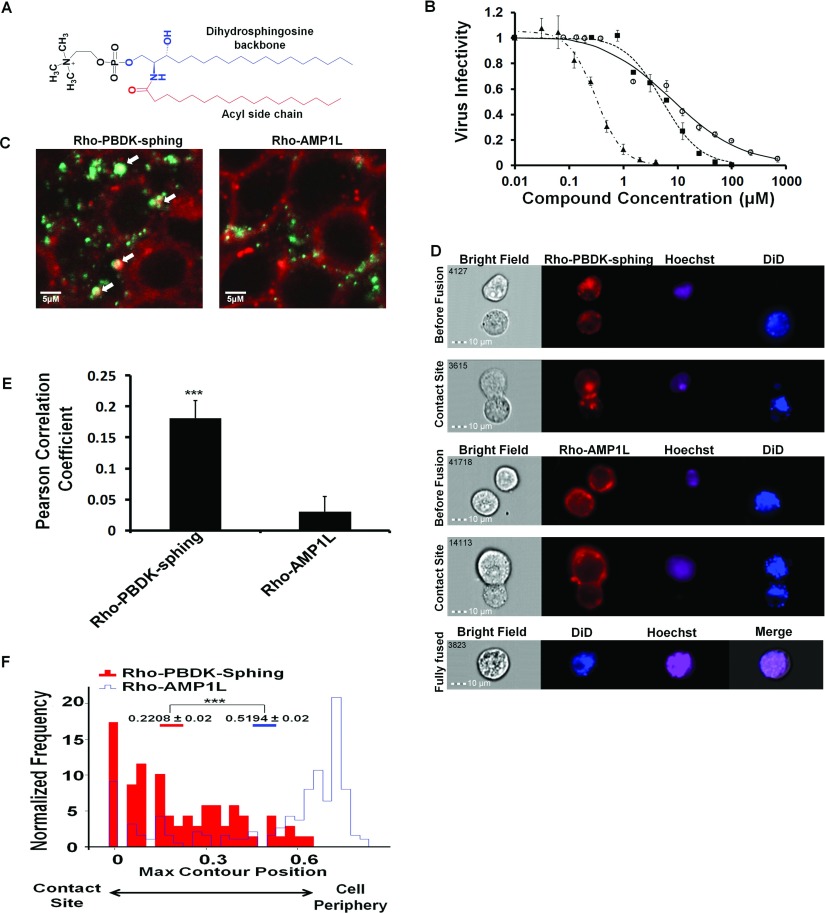
PBDK–sphing is localized to the viral entry site and to the cell–cell fusion contact site and inhibits HIV-1 infectivity (**A**) DHSM is composed of a dihydrosphingosine (sphinganine) backbone (blue), a PA acyl group (red) and a polar head group (black). (**B**) Virus–cell infectivity assay utilizing infectious virions and TZM-bl cells. PBDK (○), sphinganine (■) and PBDK–sphinganine (▲). Results are means±S.E.M. (*n*≥2). (**C**) TZM-bl cells treated with the Rho (red)-labelled PBDK–sphing and the control peptide AMP1L and infected with infectious virions (green). White arrows mark co-localization sites. Both peptides bind the cellular membrane. (**D**) PBDK–sphing is localized to the contact site during the cell–cell fusion assay. TZM-bl cells are stained with Hoechst and HL2/3 cells with DiD and full cell–cell fusion is captured as the merging of the two. Both PBDK–sphing and AMP1L are labelled with Rho. The cells were analysed via an ImageStreamX imaging flow cytometer. Doublet cells stained with either Rho–PBDK–sphing or Rho–AMP1L. Before contact the both peptides are present in the single cells. During contact the Rho–PBDK–sphing co-localizes to the contact site, whereas Rho–AMP1L does not. (**E**) Mean Pearson correlation coefficient of the fully infectious virus and each peptide. Results are means±S.E.M. (*n*≥20). ***P<0.0005. (**F**) Rho–PBDK–sphing is localized at the fusion interface whereas AMP1L binds the membrane in an unbiased ubiquitous manner. The maximum contour position feature calculates the location of the highest intensity concentration of the staining. Values closer to 1 represent the periphery of the doublet and closer to 0 the cell–cell contact site. *n*≥69, U=24745 and Z=7.579. ***P<10^−6^ as determined using the Mann–Whitney *U* test.

Various lipopeptides have been suggested to target the membrane [25,33,34] and to inhibit HIV infectivity [25,34]. One might surmise that these compounds bind to the membrane and, in the case of HIV, localize to the virus–cell contact site. We attempted to assess directly whether the conjugated peptides are targeted to the virus–cell contact site during the fusion process. To this end we first validated the anti-HIV activity of the sphingopeptide on infectious virions and on cell–cell fusion. We utilized an HIV-derived otherwise inert 15-mer peptide termed PBDK (Table 1). PBDK is derived from the PBD (pocket-binding domain) of the gp41 CHR region, which binds a conserved pocket in the NHR region [16]. The peptide was conjugated either to PA or the sphinganine base of DHSM (termed PBDK-PA and PBDK-sphing respectively) and purified using RP-HPLC (Supplementary Figure S1 at http://www.biochemj.org/bj/461/bj4610213add.htm). DHSM is composed of a lipid backbone and a polar myelin head group. The lipid backbone has a unique sphinganine base (sphing) and a saturated acyl chain, mainly PA [23] (Figure 1A). The anti-viral effect of PBDK–sphing and both components alone was investigated in a fully infectious HIV-1 infectivity assay (Figure 1B and Table 1). We found that PBDK–sphing exhibited an inhibitory concentration at 50% fusion (IC_50_) of 467 nM and an inhibitory concentration at 90% fusion (IC_90_) of 1 μM, whereas both components alone both exhibited an IC_50_ of over 1.5 μM and an IC_90_ over 6 μM (Table 1). We further examined this trend in a cell–cell fusion assay and once again PBDK–sphing exhibited a much higher inhibitory effect than any one of its components alone (Table 2). The importance of the lipid moiety was analysed by the analogue PBDK–PA. We found that PBDK–sphing showed a higher inhibitory effect than this compound (Table 1). All of the lipid conjugates were not toxic to cells in the concentration range where anti-viral activity occurred (Table 1). Overall these results suggest importance of the lipid moiety to the anti-viral activity of these compounds.

**Table 1:** Anti-HIV-1 infectivity results of PBDK-sphing and analogues A lysine residue (K) was added to the peptides for sphinganine conjugation to the C-terminus. Inhibition of viral infection: TZM-bl cells were infected with fully infectious HXB2 HIV-1 in the presence of different compounds. IC_50_ (concentration achieving 50% inhibition of infection) and IC_90_ (concentration achieving 90% inhibition of infection) values for each compound were calculated as described in the Materials and methods section. Toxicity concentrations represent a minimum 10% toxicity. TZM-bl cells were exposed to each compound and toxicity was measured using the XTT method as described in the Materials and methods section. n.d., not determined.

Designation	Sequence/formula	IC_50_ (μM)	IC_90_ (μM)	Toxicity (μM)
Sphinganine	C_18_H_39_NO_2_	>1.5	>6	>50
PBDK	TTWMEWDREINNYTK	>1.5	>6	n.d.
PBDK–sphing	TTWMEWDREINNYTK–sphing	0.467±0.09	1.09±0.07	>10
PBDK–PA	TTWMEWDREINNYTK–PA	>1.5	>6	>10
AMP1L	KLKLLKLLKLLKLLK	n.d.	n.d.	>2.5

**Table 2:** Inhibition of cell–cell fusion by PBDK–sphing, PBDK and sphinganine Inhibition of cell–cell fusion: TZM-bl target cells were mixed with HL2/3 effector cells in the presence of different compounds. IC_50_ (concentration achieving 50% inhibition of infection) and IC_90_ (concentration achieving 90% inhibition of infection) values for each compound were calculated via luciferase expression as described in the Materials and methods section.

Designation	IC_50_ (μM)	IC_90_ (μM)
Sphinganine	>1.5	>14
PBDK	>1.5	>14
PBDK–sphing	0.88±0.07	2.24±0.75

### Confocal microscopy and imaging flow cytometry reveal that HIV-1 virions and sphingopeptides co-localize on the target cell surface and at the contact site between effector and target cells

The targeting of the sphingopeptide to the viral entry site during the fusion process was assessed using infectious virions and confocal microscopy. We utilized a control peptide termed AMP1L, which binds membranes in an unbiased ubiquitous manner [35] (sequence shown in Table 1). TZM-bl cells were treated with the Rho-labelled peptides and sphingopeptides, and then treated with PKH67 fluorescently labelled infectious virions (Figure 1C). PKH67 staining efficacy was approximated close to 100% (Supplementary Figure S2 at http://www.biochemj.org/bj/461/bj4610213add.htm) and did not affect virus infectivity (Supplementary Figure S3 at http://www.biochemj.org/bj/461/bj4610213add.htm). We assessed the co-localization of each compound with virions that reached the cell surface and calculated the Pearson correlation coefficient (1 is complete localization and −1 is negative correlation) [36]. The coefficient of PBDK–sphing indicated that it was 6-fold more co-localized than AMP1L (Figure 1E). Both compounds were not toxic at relevant levels (Table 1). This suggests that sphingopeptides are localized to the sites of viral entry.

To gain further insight into the dynamics of the sphingopeptide location during the fusion process, PBDK–sphing and AMP1L were analysed in the cell–cell fusion assay in which early stages of the fusion process were captured by imaging flow cytometry (Figure 1D). The cell–cell interface is much larger than the virus–cell interface and serves as a good model for visualizing the dynamic processes of membrane fusion. In order to capture early cell–cell contacts, we utilized the ImageStreamX (Amnis) imaging flow cytometer to select the cells in such a way that coupled cells undergoing fusion (termed doublets) were separated from the general cell population, such as fused cells (Figure 1D). Analysis was carried out on doublets composed of two cell types each with a different staining of either DiD lipid dye for HL2/3 effector cells or Hoechst for TZM-bl target cells. This allowed us to choose only doublets undergoing fusion and not replicating cells of the same type. In addition, the onset of fusion by lipid dye transfer from the effector cells to target cells was evident in many of the doublets analysed and is shown in Figure 1(D) for the control AMP1L. Next, we analysed the peptide and sphingopeptide distribution in the doublets (Figure 1F). The control peptide localized more to the membrane at the periphery of the doublet rather than the fusion site. This is expected due to the ubiquitous membrane binding of AMP1L, allowing it to bind the full cell membrane. In contrast, the sphingopeptides were localized to the contact site between target and effector cells. These results illustrate the ability of sphingopeptides to be recruited to the contact sites of cells undergoing fusion.

### HIV-1 fusion protein folding is disrupted by sphingopeptides

**Figure 2:**
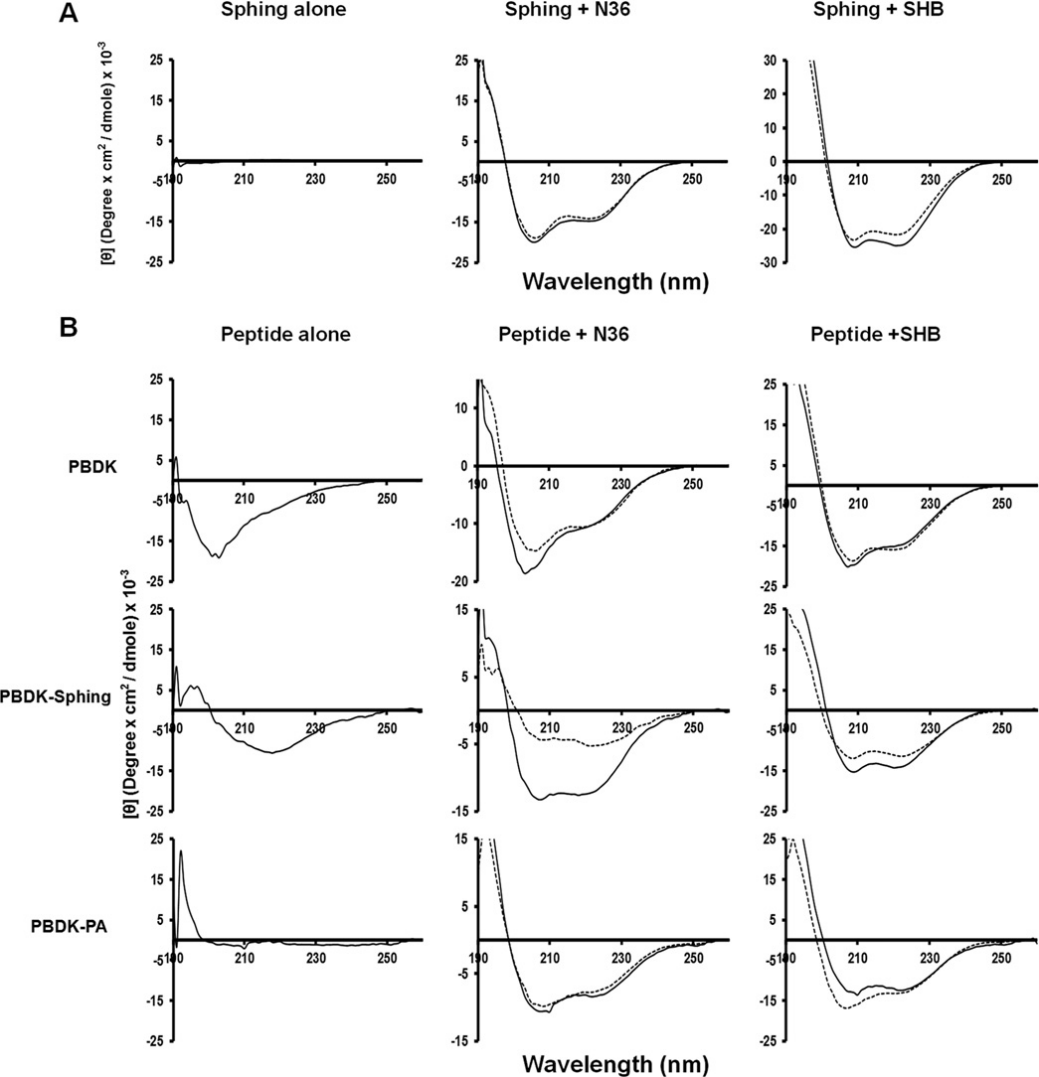
CD spectroscopy reveals that PBDK–sphing disrupts N36 assembly and SHB formation The effect of PBDK–sphing is dependent on both of its components. Peptides were measured at 10 μM in 5 mM Hepes. (**A**) CD spectra of sphinganine alone (solid line) and with the target peptide (broken line). N36 and the SHB form α-helical structures in solution. (**B**) CD spectra of the peptides alone with N36 and the SHB. The solid line indicates the non-interactive signal and the broken line indicates the interactive signal. PBDK–sphing drastically disrupts the ability of N36 and the SHB to assemble as observed by the reduction in strength of the Θ222 signal. This effect was not observed with the either component alone and when sphinganine was replaced with PA.

**Figure 3:**
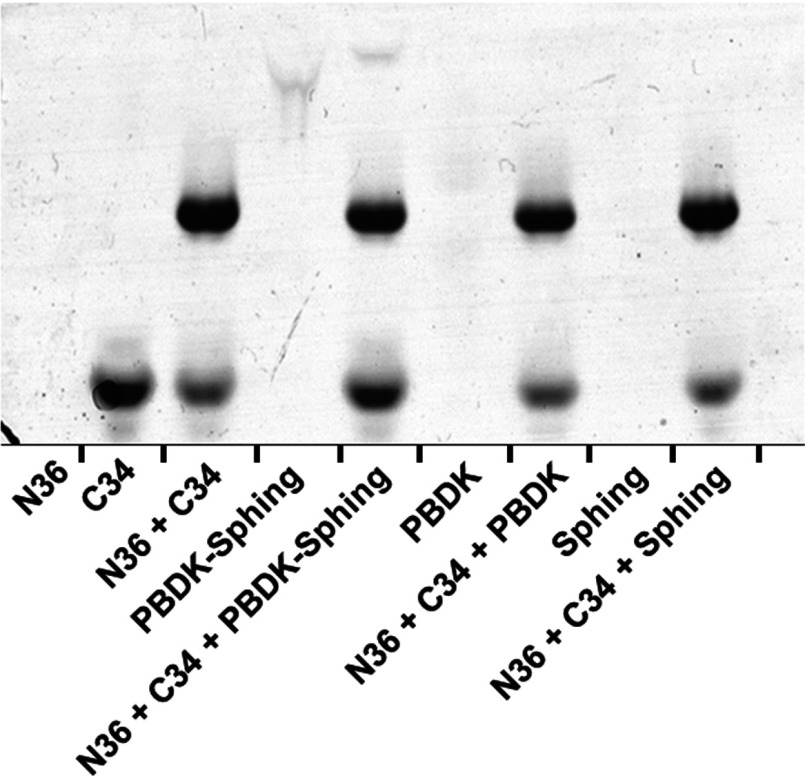
PBDK–sphinganine interferes with C34 binding to N36 Native PAGE analysis demonstrating the ability of PBDK–sphinganine to interfere with C34 binding to N36. Peptides were incubated in PBS for 30 min at 37°C, allowing peptide interactions, before being loaded on to an 18% polyacrylamide gel. Peptide concentrations were 200 μM for N36 and C34 and 100 μM for PBDK, sphinganine and PBDK–sphinganine. N36 cannot run on its own due to its isoelectric point. C34 exhibits a distinct band in the gel. When mixed together before loading, a new band appears above the C34 band, which consists of C34 bound to N36 forming the gp41 core. PBDK–sphinganine disrupts C34 binding. This is evident due to the C34 band, which grows in intensity when less binding of C34 with N36 occurs.

The SHB of gp41 is an established target for N- and C-peptide fusion inhibitors [16–20] as its complete formation is crucial for complete membrane fusion and pore formation [13–16]. We hypothesized that PBDK–sphing might affect the assembly of the SHB as PBDK is a CHR-derived peptide. Therefore the NHR domain, N36 peptide and the gp41 core structure (created by N36 together with C34, the CHR domain) were subjected to secondary structure analysis after being exposed to various PBDK–sphing analogues and PBDK–sphing itself (Figure 2). Only conjugation to the peptide of the DHSM sphinganine backbone and not the PA acyl chain (Figure 1A) conferred an active compound, which decreased the α-helical content of the N36 trimer, presumably by disrupting protein assembly (Figure 2). The findings correlate with the effect of the compound on virus infectivity (Tables 1 and 2). The data reveal two steps in which PBDK–sphing may act: (i) disruption of N36 assembly, and (ii) subsequently the disruption of gp41 core formation. Furthermore, these findings propose a mechanism that is dependent on the lipid moiety identity. Additionally the disruption of the gp41 core formation was further verified using native PAGE analysis (Figure 3). Owing to its positive charge, N36 cannot run through the gel and no band is obtained. C34, on the other hand, can run through the gel and a distinct band pattern is obtained. When C34 and N36 are mixed together a faded C34 band appears and much higher above it a second band appears which points to a larger complex, the gp41 core. The level of SHB formation can be evaluated by observing the strength of the C34 band. If C34 binding to N36 is blocked then more C34 will accumulate below the gp41 core band. Using this method we observed a distinct accumulation of C34 below the gp41 core band only when PBDK–sphinganine was added to the system. PBDK alone and sphinganine alone did not manage to disrupt gp41 core formation, which is evident due to the similarity between C34 band strength in these two compositions to the N36 and C34 with no added reagents. This corroborates the secondary structure data and strengthens the proposed mechanism that PBDK–sphing disrupts SHB formation.

### Sphinganine decreases FP-mediated lipid mixing of zwitterionic membranes

**Figure 4:**
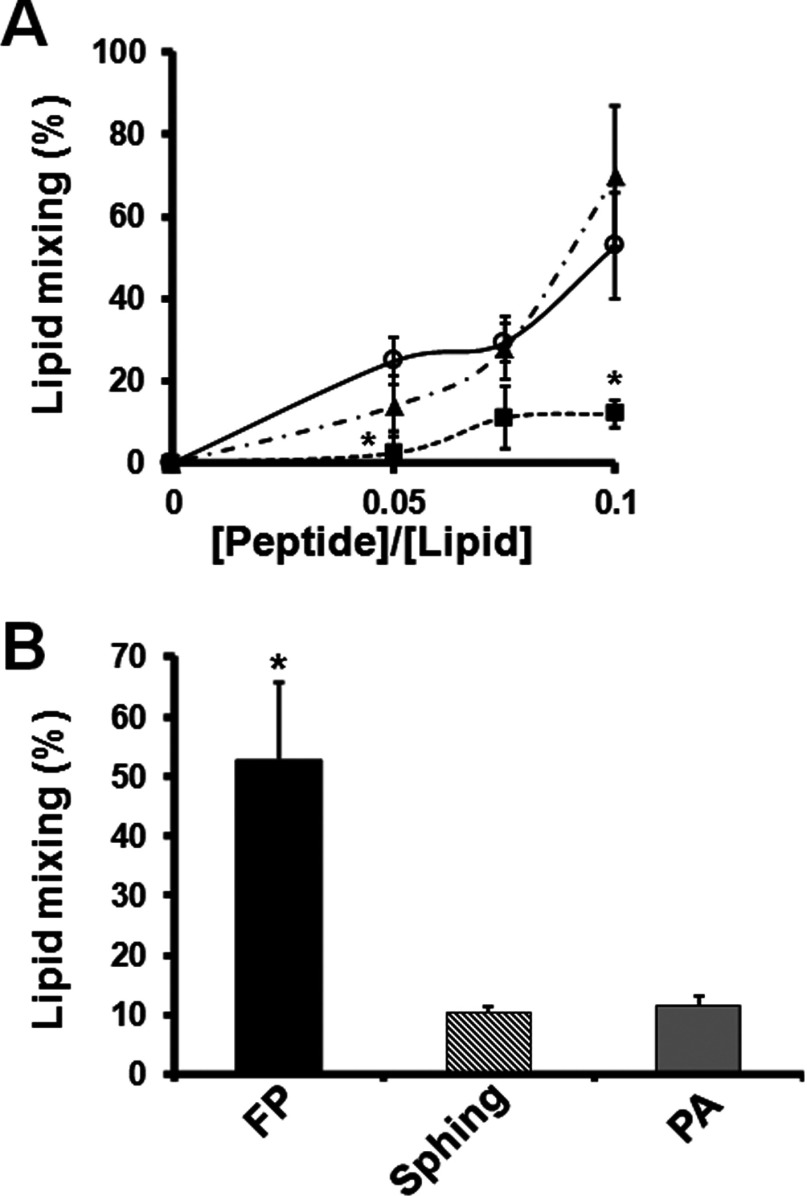
Sphinganine disrupts the lipid mixing of zwitterionic membranes induced by the FP Lipid mixing of LUVs induced by the HIV-1 FP 1–33, sphinganine, PA and Triton X-100. Peptide aliquots were added to pre-labelled LUVs and 90% unlabelled LUVs. The percentage from the maximum [defined as the 2% (v/v) increase in fluorescence of Triton X-100] was plotted against the peptide/lipid molar ratio. Results are means±S.E.M. (**A**) Dose-dependent lipid mixing by the FP. Sphinganine disrupts significantly the lipid mixing by 10 μM of the FP, whereas PA does not. FP alone (○), FP with 12.5 μM sphinganine pre-treatment (■) and FP with 12.5 μM PA pre-treatment (▲) (*n*≥2). (**B**) The ability of the treatments to induce lipid mixing was evaluated. Both lipids were added for a final concentration of 12.5 μM and the FP was added for a final concentration of 10 μM. Sphinganine and PA alone did not significantly induce lipid mixing of LUVs (*n*≥4). *P<0.05.

One of the roles of the gp41 FP during membrane fusion is to penetrate into the host cell membrane and to destabilize the membrane thereby promoting lipid mixing [11,37]. We suspected that sphinganine itself might have an effect on the mem-brane that would modulate the ability of the FP to induce lipid mixing of zwitterionic membranes, which resemble the outer leaflet of the virus and the cell's membrane. To test this possibility a lipid mixing assay was used with large unilamellar vesicles composed of PC, SM and Chol (1:1:1) that mimic the composition of ordered-domains (Figure 4). Sphinganine and PA were incorporated into the liposomes before the addition of the FP. Sphinganine and PA alone induced minor effects on lipid mixing, compared with the FP that elevated lipid mixing by 50%. Interestingly, we found that sphinganine, but not PA, incorporation into the membranes significantly reduced lipid mixing of the FP in a dose-dependent manner (Figure 4A). This could be correlated with alterations in the properties of the membrane, making it less accessible to the FP.

### CD4 diffusion through the cell membrane is disrupted by sphingopeptides

**Figure 5:**
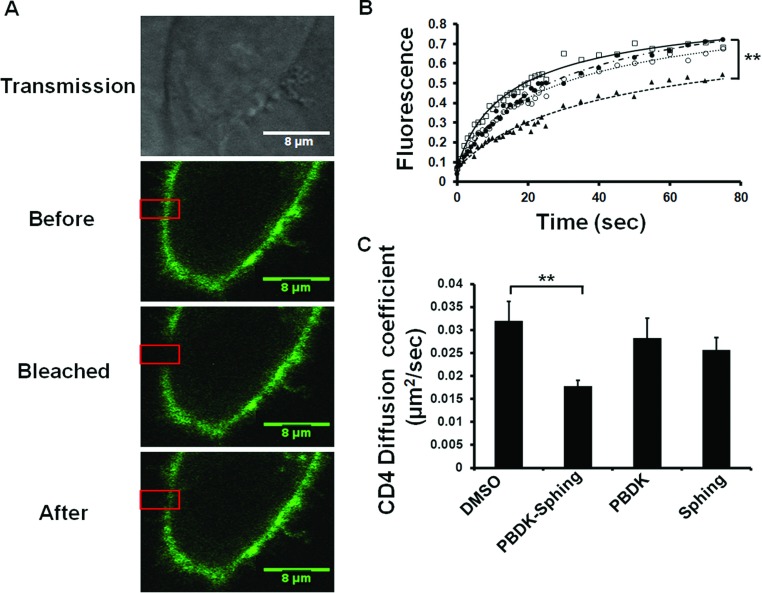
CD4 diffusion through the membrane is impaired by PBDK–sphing HeLa cells with GFP-labelled CD4 were subjected to DMSO, PBDK–sphing, PBDK alone or sphinganine (sphing) alone, and the diffusion of GFP-labelled CD4 was calculated using a FRAP assay. (**A**) Representative cell treated with PBDK–sphing and expressing GFP–CD4 under transmission light, before bleaching, bleached and after recovery of the GFP–CD4 signal. (**B**) Fluorescence recovery curves of GFP–CD4 after the four treatments to the cells: DMSO (□), PBDK–sphing (▲), PBDK alone (●) and sphing alone (○). **P<0.005 between PBDK–sphing and DMSO. (**C**) Mean calculated CD4 diffusion coefficients for the four treatments. Results are means±S.E.M. (*n*≥10). **P<0.005. PBDK–sphing was found to be the only agent that significantly inhibited the diffusion of CD4 in the membrane.

In light of the lipid mixing experiments, we suspected that sphingopeptides modulate the membrane, hence preventing receptor diffusion that is needed for the initiation of the fusion process. CD4 is a key receptor for HIV binding to the host cell and is known to concentrate at the viral-binding site [38,39]. Thus we focused on analysing CD4 mobility in target cells that were subjected to sphingopeptides and their building components (i.e. the lipid and the peptide). TZM-bl target cells expressing a GFP-labelled CD4 receptor were analysed using a FRAP experiment. Different areas in the cell membrane were bleached and the kinetics of signal recovery after photobleaching was detected to determine the diffusion coefficient factor (Figures 5A and 5B). When the cells were treated with PBDK–sphing the CD4 diffusion coefficient was found to be significantly lower as opposed to the coefficient after treatment with DMSO. Treatment with the peptide and the lipid alone did not produce the same effect (Figure 5C). Hence, attenuation of CD4 recruitment and the prevention of FP insertion to start lipid mixing support a membrane modulation mechanism, probably by stiffening the membrane.

## DISCUSSION

Various peptides derived from membrane proteins have been conjugated to lipids to exert diverse effects on biological systems within the membrane milieu [25,33,34]. These emerging studies advanced the understanding of membrane modulation processes that are mediated by membrane proteins. To date, it was belie-ved that lipid conjugation only anchors the peptides to the membrane thereby enriching their local concentration, thus allowing peptide binding to the membrane protein affecting its conformation. In the present study, we report a novel mechanism in which the lipid and the peptide disrupt early as well as late membrane fusion events by a joint action on the fusion protein and on the membrane.

CD4 recruitment to the HIV–cell binding site occurs at an early stage of the fusion process [29,38,39], later followed by the gp41FP insertion into the host cell membrane, which destabilizes the membrane [37,40]. Sphinganine alone and the peptide alone did not affect CD4 diffusion, as opposed to the sphingopeptides. Sphinganine alone is rapidly up taken by the cells [23] and might traffic away from the fusion site. Since the peptide itself did not affect the diffusion ability of CD4, it is reasonable that when conjugated to sphinganine the peptide holds the lipid at the plasma membrane allowing the restriction of receptor lateral diffusion. This is strengthened by the ability of sphingopeptides to strongly bind the membrane and still inhibit virus entry after extensive cell washing [25]. The observed impairment of CD4 diffusion is due to sphingopeptide modulation of the physical properties of the membrane possibly by stiffening [41]. Another functional consequence supporting membrane stiffening is the prevention of zwitterionic lipid mixing by the FP when the membrane was treated with sphinganine. Modulation of membrane properties by alterations in the lipid composition of the cell and viral membrane before infection by robust treatments has been shown to disrupt both viral infectivity and cell–cell fusion [2,6,42,43]. Interestingly, increased levels of saturated DHSM in the cell membrane disrupt HIV infectivity [41]. Our results suggest that sphinganine rather than fatty acid (PA) is the active lipid backbone of DHSM. Moreover, the polar hydroxy groups located in the sphinganine backbone interact with neighbouring sphingolipid-containing hydroxy groups through hydrogen bonds [23], hence stabilizing DHSM interactions. In addition, lipid saturation in the ER (endoplasmic reticulum) modulates the function of ER-resident membrane proteins [44], implying a common mechanism in diverse lipid systems.

In the later HIV-mediated membrane fusion stages, sphingopeptides act through interference of gp41 assembly. The interference with gp41 assembly by peptides derived from the CHR region, as shown in the present study, involves competing with the endogenous CHR region's binding to the NHR region by a dominant-negative mechanism thereby preventing core formation [14,16]. Normally, these hybrid inhibitory interactions form a stabilized complex with the NHR that increase its α-helical structure [14,16]. In the present paper, we report an unusual disruption mechanism of the NHR region assembly only when sphinganine and the peptide were added as a single molecule. This accounts for the reduction observed in core formation. The antiviral properties of the sphinganine backbone are intriguing, as attenuated HIV propagation by lipids would pose a high hurdle for the virus to cross through mutational processes.

An interesting property of the sphinganine lipidated peptides is their ability to co-localize to the viral–cell contact site and cell–cell fusion site. This ability might be due to a preference of the sphinganine moiety to incorporate into membrane-ordered domains. These domains are enriched with sphingomyelin [3] and mediate HIV entry and exit [4–6]. Sphinganine can interact with the sphingolipid backbone through hydrogen bonds and it can also form condensed regions with Chol [24]. In addition, the peptide itself can interact with the HIV envelope thus elevating the concentration of the sphingopeptides at the contact sites.

By subjecting the sphinganine to HIV peptide conjugation, we observed a complex relationship that combines the unique properties of sphinganine together with the inherent properties of the peptide. The specific effects that these lipopeptides have on the membrane as well as their exact orientation at the membrane interface still needs future investigation. Nevertheless, these findings shed light on the intricacies of sphingopeptide activity and reveal a partnership that can modulate fusion protein functionality and in addition modulate the cell membrane as well as membrane-embedded receptors. Moreover, recent studies show elevation of DHSM in HIV-1 host cells (primarily MT-4 cells) and their derived virions [45] and stress the importance of the lipid environment to membrane protein function [46]. Consequently, our findings may contribute in understanding the function of DHSM situated in the HIV-1 lipidome and host–cell membrane.

## Online data

Supplementary data

## References

[1] Rawat S. S., Viard M., Gallo S. A., Blumenthal R., Puri A. (2006). Sphingolipids, cholesterol, and HIV-1: a paradigm in viral fusion. Glycoconj. J..

[2] Liao Z., Cimakasky L. M., Hampton R., Nguyen D. H., Hildreth J. E. (2001). Lipid rafts and HIV pathogenesis: host membrane cholesterol is required for infection by HIV type 1. AIDS Res. Hum. Retroviruses.

[3] Simons K., Ikonen E. (1997). Functional rafts in cell membranes. Nature.

[4] Nguyen D. H., Hildreth J. E. (2000). Evidence for budding of human immunodeficiency virus type 1 selectively from glycolipid-enriched membrane lipid rafts. J. Virol..

[5] Ono A., Freed E. O. (2001). Plasma membrane rafts play a critical role in HIV-1 assembly and release. Proc. Natl. Acad. Sci. U.S.A..

[6] Viard M., Parolini I., Sargiacomo M., Fecchi K., Ramoni C., Ablan S., Ruscetti F. W., Wang J. M., Blumenthal R. (2002). Role of cholesterol in human immunodeficiency virus type 1 envelope protein-mediated fusion with host cells. J. Virol..

[7] Simons K., Warren G. (1984). Semliki Forest virus: a probe for membrane traffic in the animal cell. Adv. Protein Chem..

[8] Simons K., Garoff H. (1980). The budding mechanisms of enveloped animal viruses. J. Gen. Virol..

[9] Kielian M., Helenius A., Schlesinger S., Schlesinger M. J. (1986). The Togaviridae and Flaviviridae.

[10] White J. M. (1990). Viral and cellular membrane fusion proteins. Annu. Rev. Physiol..

[11] Blumenthal R., Clague M. J., Durell S. R., Epand R. M. (2003). Membrane fusion. Chem. Rev..

[12] Finzi A., Xiang S. H., Pacheco B., Wang L., Haight J., Kassa A., Danek B., Pancera M., Kwong P. D., Sodroski J. (2010). Topological layers in the HIV-1 gp120 inner domain regulate gp41 interaction and CD4-triggered conformational transitions. Mol. Cell.

[13] Buzon V., Natrajan G., Schibli D., Campelo F., Kozlov M. M., Weissenhorn W. (2010). Crystal structure of HIV-1 gp41 including both fusion peptide and membrane proximal external regions. PLoS Pathog..

[14] Gallo S. A., Finnegan C. M., Viard M., Raviv Y., Dimitrov A., Rawat S. S., Puri A., Durell S., Blumenthal R. (2003). The HIV Env-mediated fusion reaction. Biochim. Biophys. Acta.

[15] Melikyan G. B., Markosyan R. M., Hemmati H., Delmedico M. K., Lambert D. M., Cohen F. S. (2000). Evidence that the transition of HIV-1 gp41 into a six-helix bundle, not the bundle configuration, induces membrane fusion. J. Cell Biol..

[16] Chan D. C., Chutkowski C. T., Kim P. S. (1998). Evidence that a prominent cavity in the coiled coil of HIV type 1 gp41 is an attractive drug target. Proc. Natl. Acad. Sci. U.S.A..

[17] Furuta R. A., Wild C. T., Weng Y., Weiss C. D. (1998). Capture of an early fusion-active conformation of HIV-1 gp41. Nat. Struct. Biol..

[18] Munoz-Barroso I., Durell S., Sakaguchi K., Appella E., Blumenthal R. (1998). Dilation of the human immunodeficiency virus-1 envelope glycoprotein fusion pore revealed by the inhibitory action of a synthetic peptide from gp41. J. Cell Biol..

[19] Wexler-Cohen Y., Shai Y. (2009). Membrane-anchored HIV-1 N-heptad repeat peptides are highly potent cell fusion inhibitors via an altered mode of action. PLoS Pathog..

[20] Liu S., Lu H., Niu J., Xu Y., Wu S., Jiang S. (2005). Different from the HIV fusion inhibitor C34, the anti-HIV drug Fuzeon (T-20) inhibits HIV-1 entry by targeting multiple sites in gp41 and gp120. J. Biol. Chem..

[21] Brugger B., Glass B., Haberkant P., Leibrecht I., Wieland F. T., Krausslich H. G. (2006). The HIV lipidome: a raft with an unusual composition. Proc. Natl. Acad. Sci. U.S.A..

[22] Epand R. M. (2003). Cholesterol in bilayers of sphingomyelin or dihydrosphingomyelin at concentrations found in ocular lens membranes. Biophys. J..

[23] Goni F. M., Alonso A. (2006). Biophysics of sphingolipids I. Membrane properties of sphingosine, ceramides and other simple sphingolipids. Biochim. Biophys. Acta.

[24] Kuikka M., Ramstedt B., Ohvo-Rekila H., Tuuf J., Slotte J. P. (2001). Membrane properties of D-erythro-*N*-acyl sphingomyelins and their corresponding dihydro species. Biophys. J..

[25] Ashkenazi A., Viard M., Unger L., Blumenthal R., Shai Y. (2012). Sphingopeptides: dihydrosphingosine-based fusion inhibitors against wild-type and enfuvirtide-resistant HIV-1. FASEB J..

[26] Merrifield R. B., Vizioli L. D., Boman H. G. (1982). Synthesis of the antibacterial peptide cecropin A (1–33). Biochemistry.

[27] Wexler-Cohen Y., Ashkenazi A., Viard M., Blumenthal R., Shai Y. (2010). Virus–cell and cell–cell fusion mediated by the HIV-1 envelope glycoprotein is inhibited by short gp41 N-terminal membrane-anchored peptides lacking the critical pocket domain. FASEB J..

[28] George T. C., Fanning S. L., Fitzgerald-Bocarsly P., Medeiros R. B., Highfill S., Shimizu Y., Hall B. E., Frost K., Basiji D., Ortyn W. E. (2006). Quantitative measurement of nuclear translocation events using similarity analysis of multispectral cellular images obtained in flow. J. Immunol. Methods.

[29] Finnegan C. M., Rawat S. S., Cho E. H., Guiffre D. L., Lockett S., Merrill A. H., Blumenthal R. (2007). Sphingomyelinase restricts the lateral diffusion of CD4 and inhibits human immunodeficiency virus fusion. J. Virol..

[30] Popik W., Alce T. M. (2004). CD4 receptor localized to non-raft membrane microdomains supports HIV-1 entry. Identification of a novel raft localization marker in CD4. J. Biol. Chem..

[31] Lippincott-Schwartz J., Presley J. F., Zaal K. J., Hirschberg K., Miller C. D., Ellenberg J. (1999). Monitoring the dynamics and mobility of membrane proteins tagged with green fluorescent protein. Methods Cell Biol..

[32] McAuliffe M. J., Lalonde F. M., McGarry D., Gandler W., Csaky K., Trus B. L. (2005). Medical image processing, analysis and visualization in clinical research. Proceeding CBMS ‘01 Proceedings of the Fourteenth IEEE Symposium on Computer-Based Medical Systems.

[33] Rajendran L., Schneider A., Schlechtingen G., Weidlich S., Ries J., Braxmeier T., Schwille P., Schulz J. B., Schroeder C., Simons M. (2008). Efficient inhibition of the Alzheimer's disease β-secretase by membrane targeting. Science.

[34] Ingallinella P., Bianchi E., Ladwa N. A., Wang Y. J., Hrin R., Veneziano M., Bonelli F., Ketas T. J., Moore J. P., Miller M. D., Pessi A. (2009). Addition of a cholesterol group to an HIV-1 peptide fusion inhibitor dramatically increases its antiviral potency. Proc. Natl. Acad. Sci. U.S.A..

[35] Ashkenazi A., Faingold O., Kaushansky N., Ben-Nun A., Shai Y. (2013). A highly conserved sequence associated with the HIV gp41 loop region is an immunomodulator of antigen-specific T cells in mice. Blood.

[36] Bolte S., Cordelieres F. P. (2006). A guided tour into subcellular colocalization analysis in light microscopy. J. Microsc..

[37] Gallaher W. R. (1987). Detection of a fusion peptide sequence in the transmembrane protein of human immunodeficiency virus. Cell.

[38] Hubner W., McNerney G. P., Chen P., Dale B. M., Gordon R. E., Chuang F. Y., Li X. D., Asmuth D. M., Huser T., Chen B. K. (2009). Quantitative 3D video microscopy of HIV transfer across T cell virological synapses. Science.

[39] McDonald D., Wu L., Bohks S. M., KewalRamani V. N., Unutmaz D., Hope T. J. (2003). Recruitment of HIV and its receptors to dendritic cell–T cell junctions. Science.

[40] Carr C. M., Kim P. S. (1993). A spring-loaded mechanism for the conformational change of influenza hemagglutinin. Cell.

[41] Vieira C. R., Munoz-Olaya J. M., Sot J., Jimenez-Baranda S., Izquierdo-Useros N., Abad J. L., Apellaniz B., Delgado R., Martinez-Picado J., Alonso A. (2010). Dihydrosphingomyelin impairs HIV-1 infection by rigidifying liquid-ordered membrane domains. Chem. Biol..

[42] St Vincent M. R., Colpitts C. C., Ustinov A. V., Muqadas M., Joyce M. A., Barsby N. L., Epand R. F., Epand R. M., Khramyshev S. A., Valueva O. A. (2010). Rigid amphipathic fusion inhibitors, small molecule antiviral compounds against enveloped viruses. Proc. Natl. Acad. Sci. U.S.A..

[43] Wolf M. C., Freiberg A. N., Zhang T., Akyol-Ataman Z., Grock A., Hong P. W., Li J., Watson N. F., Fang A. Q., Aguilar H. C. (2009). A broad-spectrum antiviral targeting entry of enveloped viruses. Proc. Natl. Acad. Sci. U.S.A..

[44] Volmer R., van der Ploeg K., Ron D. (2012). Membrane lipid saturation activates endoplasmic reticulum unfolded protein response transducers through their transmembrane domains. Proc. Natl. Acad. Sci. U.S.A..

[45] Lorizate M., Sachsenheimer T., Glass B., Habermann A., Gerl M. J., Krausslich H. G., Brugger B. (2013). Comparative lipidomics analysis of HIV-1 particles and their preducer cell membrane in different cell lines. Cell. Microbiol.

[46] Ernst A. M., Brugger B. (2013). Sphingolipids as modulators of membrane proteins. Biochim. Biophys. Acta.

