# Supplementary material for: Early and late HIV-1 membrane fusion events are impaired by sphinganine lipidated peptides that target the fusion site
Source: Biochem J. 2014 Jun 26;461(Pt 2):213–22. doi: 10.1042/BJ20140189 (PMC4072049; doi:10.1042/BJ20140189)
Supplement: Supplementary data [file bj4610213add.pdf]

BJ Cell

Biochem. J. (2014) **461**, 213–222 (Printed in Great Britain) doi:10.1042/BJ20140189

SUPPLEMENTARY ONLINE DATA

Early and late HIV-1 membrane fusion events are impaired by sphinganine lipidated peptides that target the fusion site

Yoel A. KLUG*, Avraham ASHKENAZI*[1], Mathias VIARD†‡, Ziv PORAT§, Robert BLUMENTHAL† and Yechiel SHAI*[2]

*Department of Biological Chemistry, Weizmann Institute of Science, Rehovot 7610001, Israel
†Section on Membrane Structure and Function, Basic Research Laboratory, Center for Cancer Research, National Cancer Institute, National Institutes of Health, Frederick, MD 21702, U.S.A.
‡Basic Science Program, Leidos Biomedical Research, NCI Center for Cancer Research, Frederick National Laboratory for Cancer Research, Frederick, MD 21702, U.S.A.
§Flow Cytometry Unit, Department of Biological Services, Weizmann Institute of Science, Rehovot 7610001, Israel

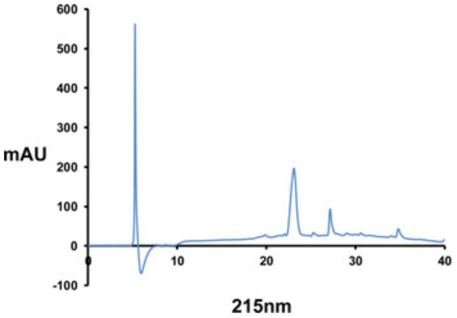

Figure S1 PBDK–sphing RP-HPLC profile post-purification

PBDK–sphing was purified using RP-HPLC as described in the Materials and methods section of the main text. To validate purity, 20 μg of the purified peptide stock was run at an acetonitrile gradient of 10–90 % for 40 min on a C_4 column. All peaks were validated as the same compound by ESI analysis. The resulting peaks are most probably oligomers of the peptide since collecting and then re-injecting each peak results in the above spectrum. The reading was done at 215 nm with a reference of 650 nm. mAU, milli-absorption units.

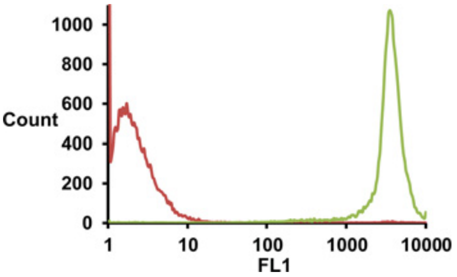

Figure S2 PKH67 staining on TZM-bl cells reveals a high staining efficacy

In order to assess PKH67 (excitation, 490 nm and emission, 504 nm) general staining efficacy stained (green) and unstained (red) TZM-bl cells were subjected to FACS analysis. A close to 100 % staining efficacy was observed for the PKH67 evident by the lack of unstained cells in the population that underwent staining.

Received 11 February 2014/23 April 2014; accepted 25 April 2014
Published as BJ Immediate Publication 25 April 2014, doi:10.1042/BJ20140189

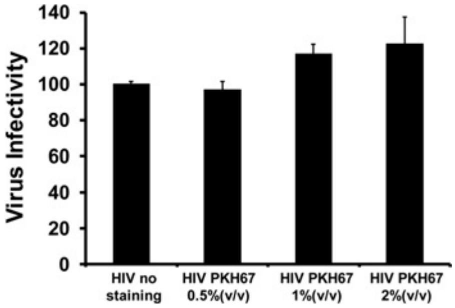

Figure S3 PKH67 staining does not affect HIV-1 infectivity

HIV-1 virions were stained with PKH67 at the noted doses and subjected to a virus–cell infectivity assay with TZM-bl cells. At the evaluated doses no significant change of infectivity was observed. The 2 % (v/v) dose was eventually used for virus labelling. The results are normalized to the infectivity of the unstained virions. Results are means ± S.E.M. ($n = 2$). $P > 0.05$ for all columns in relation to unstained HIV.

[1] Present address: Cambridge Institute for Medical Research, University of Cambridge, Cambridge CB2 0XY, U.K.
[2] To whom correspondence should be addressed (email Yechiel.Shai@weizmann.ac.il).

© 2014 The Author(s) The author(s) has paid for this article to be freely available under the terms of the Creative Commons Attribution Licence (CC-BY) (http://creativecommons.org/licenses/by/3.0/) which permits unrestricted use, distribution and reproduction in any medium, provided the original work is properly cited.

Biochemical Journal www.biochemj.org
